# Behavioral Changes Under Levetiracetam Treatment in Dogs

**DOI:** 10.3389/fvets.2020.00169

**Published:** 2020-04-03

**Authors:** Johannes Roland Erath, Jasmin Nicole Nessler, Franziska Riese, Enrice Hünerfauth, Karl Rohn, Andrea Tipold

**Affiliations:** ^1^Department of Small Animal Medicine and Surgery, University of Veterinary Medicine, Hanover, Germany; ^2^Institute for Biometry, Epidemiology and Information Processing, University of Veterinary Medicine, Hanover, Germany

**Keywords:** canine, seizure, epilepsy, levetiracetam, behavior, side effect

## Abstract

In veterinary medicine levetiracetam (LEV) is a well-tolerated antiepileptic drug (AED) with only mild to moderate side effects. Behavioral changes are rarely reported in animals. In contrast, in human medicine the impact of LEV on behavior has frequently been described. Since in the Clinic for Small Animals at the University of Veterinary Medicine Hannover single canine patients were observed with behavioral abnormalities after LEV treatment, it was hypothesized that levetiracetam induces behavioral changes or causes an intensifying of pre-existing behavioral abnormalities in dogs with epileptic seizures. This monocentric retrospective study evaluated the incidence of behavioral changes in epileptic dogs treated with the antiepileptic drug LEV based on information obtained in a questionnaire completed by dog owners. Eighty-four client-owned dogs with recurrent seizures receiving LEV as monotherapy, add on treatment or pulse therapy met inclusion criteria. Approximately half of the dogs in the study population were reported to have preexisting behavioral changes before treatment with LEV, and some of these dogs were reported to experience a worsening of behavioral changes (14/44) or the emergence of new behaviors after initiation of LEV therapy (4/44). One quarter of the dogs without pre-existing behavioral abnormalities developed behavioral changes associated with the administration of LEV (10/40). Based on these results, the authors conclude that behavioral changes can occur in dogs being administered LEV, and this should be taken into consideration when discussing treatment options with owners.

## Introduction

Seizures are a common cause for presentation in veterinary practices (1). This symptom may occur due to chronic brain diseases such as structural or idiopathic epilepsy (2, 3). It might be also induced by an underlying systemic metabolic or toxic disease (3). Currently, phenobarbital, imepitoin and as add-on medication potassium bromide are licensed in Europe for the treatment of canine epilepsy (4). Antiepileptic drugs (AEDs) of the newer generation applied in human medicine can only be used after applying the cascade system. One of these AEDs is LEV. This medication is used in case of severe side effects of the licensed products or as add-on treatment in refractory epileptic seizures (5, 6). Additionally, LEV is given, when cluster seizures, status epilepticus or myoclonus epilepsy occurs (6, 7). LEV is well-tolerated in dogs with only mild to moderate adverse effects (4, 5, 8). In addition, clinical chemistry, hematology, or urinalysis values are not changed under LEV treatment (9). The most frequently reported side effects in veterinary medicine are vomiting, sedation, and ataxia (10). However, new findings in human medicine, especially in pediatric research, revealed an occurrence of behavioral side effects due to LEV application (11). Behavioral side effects are more likely to occur in cases of pre-existing behavioral problems in humans (12–14). Currently, the dosage and its influence on human behavior are not known (14–16). To the knowledge of the authors, there are no studies focusing only on behavioral abnormalities as side effect caused by LEV application in dogs. Therefore, the occurrence of behavioral changes after LEV treatment may be underestimated in veterinary medicine. Pets are of great importance in the life's of many people (17). Often they are seen as family members (18). Due to this high level of social interaction between pets and humans, behavioral problems of a dog may have negative impact on the dog-owner relationship (19). Moreover, dogs' behavioral abnormalities are affecting its quality of life, which is of great importance to the owners (20).

Since in the Department for Small Animal Medicine and Surgery of the University of Hannover, single canine patients with behavioral abnormalities after LEV application were observed, the current study aims to elucidate the occurrence of behavioral changes after administration of this AED. Therefore, a validated standardized questionnaire was used to examine patients behavior. The hypothesis of this study is, that LEV treatment induces behavioral changes or causes an intensifying of pre-existing behavioral abnormalities in dogs with epileptic seizures.

## Materials and Methods

### Study Population/Data Collection

Study members for this retrospective study were client-owned dogs of the Department for Small Animal Medicine and Surgery of the University of Hannover receiving levetiracetam within the last 5 years. Medical records were reviewed between January 2013 and December 2018. Data acquisition was performed via the patient management system (“easy vet,” VetZGmbH, Isernhagen, Germany) using the search term “levetiracetam.”

**Figure 1:**
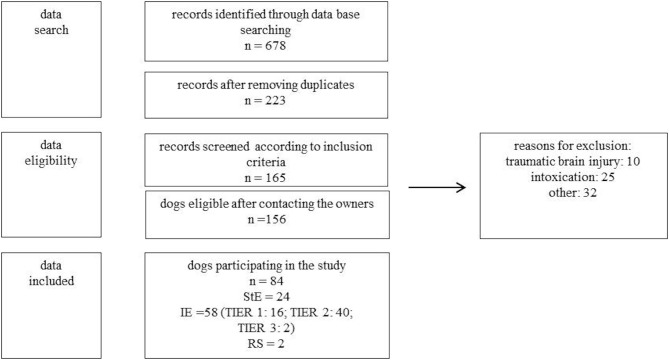
Study protocol for data analysis. n, number of dogs; StE, structural epilepsy; IE, idiopathic epilepsy (TIER 1,2,3); RS, reactive seizures; other, other reasons for exclusion (e.g., incomplete data, movement disorder, owner refused participation).

Using this search term 678 records were found. After removing duplicates resp. records of dogs with multiple consultations, 223 client-owned dogs were eligible. After review of the 223 patient files, 156 patients met inclusion criteria (Figure 1).

The following inclusion criteria were used: seizures were caused by idiopathic epilepsy, structural epilepsy or reactive seizures. Dogs with seizures due to “intoxication” or “traumatic brain injury” had to be excluded due to the fact that LEV was only given once to manage severe seizures or even preventive. Further, dogs with movement disorders, incomplete data, or a lacking of owner approval for participating in the study had to be excluded as well. Diagnoses were established according to the guidelines of the “International veterinary epilepsy task force” (3, 21). In total, owners of 84 dogs were included in the study protocol for data analysis (Figure 1).

Behavior of the dogs was retrospectively evaluated before and after LEV treatment. Therefore, a new questionnaire was created, based on three previous validated surveys (22–24). The described surveys, were, if necessary, translated into German. The questionnaire was divided into general questions concerning the data protection declaration, signalment, clinical signs and the behavior of the dog before and during antiseizure treatment with LEV. Furthermore, at the end of the questionnaire the translated questions of the two validated surveys by Hsu and Serpell (22) and Shihab et al. (23) had to be completed to verify the previous answers. This was achieved by completing identical standardized behavioral questions concerning dogs behavior before and after LEV treatment. In addition, the answers of the owners were compared with the medical records including the neurological examination to gather additional information. To compare the answers with the medical database, each patient received an access code to fulfill current data protection regulation and ethical approval of the University. The questionnaires were filled in electronically (*n* = 33) via LimeSurvey Version 2.05+ (LimeSurveyGmbH, Hamburg, Germany), on paper in clinics during a follow up examination or at home (*n* = 49). Additionally, telephone interviews were conducted if preferred by the owners (*n* = 2).

Questionnaires were collected from August 2018 to December 2018. The median time between initiation of levetiracetam treatment to owner response was 22.15 months (range, 0.4–75.7 months).

The questionnaire is published under supplementary material in German (Supplementary Figure 1) and English language (Supplementary Figure 2).

After analysis of the completed questionnaires, the dogs were divided into two groups: dogs with a history of behavioral abnormalities before LEV treatment and dogs without any pre-existing behavioral changes to minimize the risk of biasing the results and misinterpreting pre-existing behavioral abnormalities as drug induced. In addition, the effect of age at onset of the first epileptic seizure was set at 2 years in the current study to examine the impact on behavioral change in juvenile and adolescent dogs.

### Statistical Analysis

To evaluate the influence of epileptic seizure frequency, sex, epilepsy etiology, age at onset of the first epileptic seizure and polypharmacy on occurrence of behavioral changes after LEV application, statistical analysis was performed using a commercial statistical software (Statistical Analysis System for Windows SAS®, version 9.4 by using the SAS® Enterprise Guide® version 7.15 Client (SAS Institute Inc., Cary, North Carolina, USA). Model-residuals of quantitative data were checked for normal distribution using Kolmogorov-Smirnov test and visual assessment of QQ plots. For data which were not normally distributed, median (m) and minimum-maximum, (min-max) are described. Since the assumption on normal distribution of quantitative parameters was rejected, non-parametric methods (Wilcoxon two sample Test, Kruskall-Wallis-Test) were used. To evaluate qualitative data a Fisher exact test was implemented. A *p* < 0.05 was considered to be significant.

## Results

### Study Population

Eighty-four dogs met the inclusion criteria. Fiftyeight dogs had idiopathic epilepsy (TIER 1: 16; TIER 2: 40; TIER 3: 2, IE), 24 structural epilepsy (neoplasia: 7; meningoencephalitis of unknown origin: 5; degenerative encephalopathy: 2; intracranial infarct: 2; myoclonus epilepsy: 2; post traumatic epilepsy: 2; presumed neoplasia: 2; hydrocephalus: 1; Aspergillosis: 1, StE) and two reactive seizures (hepatoencephalic syndrome, RS) (Figure 1). Median age of the dogs was six years (range 0.33–16 years). In the study participated twelve intact females, 17 spayed females, 27 intact males, and 28 neutered males. Median weight of the dogs was 20 kg (range 3.5–68 kg). Breeds included 24 crossbreeds, five Siberian Huskies, four Australian Shepherds, three Pug dogs, three Miniature Schnauzers, three Golden Retrievers. Two of each of the following breeds: Wire-Haired Dachshund, Miniature Bull Terrier, Jack Russel Terrier, Labrador Retriever, Boxer, West Highland White Terrier, Rhodesian Ridgeback, Magyar Vizsla, German Shepherds, Yorkshire Terrier and one of the following breeds: Chihuahua, Dogo Argentino, Cao de Serra de Aires, Fox Terrier, French Bulldog, Istrian Bracke, Landseer, German Spitz, Continental Bulldog, German shorthaired pointer, Pomeranian, Perro de Agua espagnol, Sheep Poodle, English Setter, Entlebucher Mountain Dog, German Pinscher, Pyrenean Mountain Dog, Sheltie, Puggle, Miniature American Shepherd, Beagle and German Bracke.

### Patient Groups

Group 1: Dogs with pre-existing behavioral abnormalities before LEV treatment (*n* = 44).

Group 2: Dogs without behavioral abnormalities before LEV treatment (*n* = 40).

In total 43 (51%) of the 84 dogs developed a change or an intensifying of their pre-existing behavior abnormalities after LEV treatment (Table 1).

**Table 1:** Behavioral changes after levetiracetam application in the whole study population.

**Behavioral change**	**Number of dogs *n* = 84 (100%)**
	IE = 58
	StE = 24
	RS = 2
Negative behavioral change	**14 (16.7%)**
	IE = 12
	StE = 1
	RS = 1
Intensification of pre-existing behavioral abnormalities	**14 (16.7%)**
	IE = 5
	StE = 8
	RS = 1
Positive behavioral change	**15 (17.9%)**
	IE = 8
	StE = 7
No change in behavior	**41 (48.8%)**
	IE = 33
	StE = 8

The changes are further described in the next paragraphs:

#### Dogs With Pre-existing Behavioral Abnormalities Before LEV Treatment (Group 1; n = 44)

Dogs with a history of behavioral abnormalities had a median age of 6 years (range, 1–16 years) and a median weight of 21 kg (range 4.5–51 kg). Canine patients had either IE (*n* = 25), StE (*n* = 18), or RS (*n* = 1). A statistical significant association for pre-existing behavior abnormalities to epilepsy etiology was found within group one (*p* = 0.017). Considering the distribution of the seizure cause within both groups, 75% of the dogs with structural epilepsy were within group one. Besides, study participants of group one consisted of six intact females, nine spayed females, 14 intact males and 15 neutered males. The occurrence of pre-existing behavioral abnormalities was gender independent of the examined dogs (*p* > 0.05). In seven dogs LEV was given as monotherapy for a median duration of 9 months (range, 1.5–24 months), receiving a median dosage of 24.2 mg/kg three times daily (range, 12.7–28.8 mg/kg TID). In 13 dogs LEV was applied as add-on treatment to other AEDs for a median time of 4 months (range, 0.25–36 months). In 11 of these 13 dogs the median dosage was 24.6 mg/kg TID (range, 13.2–50 mg/kg TID). The remaining two dogs were treated with a median dosage of 31.9 mg/kg four times daily (range, 26.3–37.5 mg/kg QID). Moreover, 24 dogs were treated with LEV pulse therapy (25). Either the pulse protocol was administered as described by Packer et al. (25) or LEV was initially given at 60 mg/kg, followed by a period of 20 mg/kg every 8 h for 3 days. After that, LEV was given at 10 mg/kg TID for further 3 days. The protocol was terminated with a dosage of 10 mg/kg every 12 h for 2 days. Only nine dogs were seizure free at the time of the study. Five of these nine seizure free dogs were treated with long-term LEV monotherapy. Thirty-four dogs had still recurrent seizures and in one dog no information about seizure occurrence was available. All owners were asked to report seizure frequency and type of seizures of their dogs to get an impression on seizure severity and to avoid misinterpretation of results. A seizure frequency of at least one seizure ≤4 weeks was reported in 30 dogs. Seven dogs had seizures every 4–12 weeks. Three dogs had no pattern of seizure recurrence and in four dogs no data about the seizure frequency were available. Forty-two dogs had generalized seizures, while two dogs showed focal seizures. Thirty-four dogs were suffering from cluster seizures. In 20 dogs the first seizure event occurred during their first 2 years of life, 24 dogs were older than 2 years at the time of first seizure occurrence. Moreover, 43 dogs were treated with AEDs as permanent therapy. Twenty-four dogs were treated with one AED, 13 dogs with two AEDs, six dogs with three AEDs. One dog did not receive any long-term antiepileptic therapy due to severe side effects and was therefore only treated with LEV pulse therapy.

Of the 44 dogs, 10/44 dog owners graded the behavioral change after LEV treatment in a positive way. Positive changes were stated as a decrease in negative behavior in comparison to the status before treatment or if the owner reported a positive behavioral effect after LEV administration. Increased activity (*n* = 4), more energy (*n* = 4), calmer mood (*n* = 3), higher tolerance to environmental stress (*n* = 3), increased obedience (*n* = 1), and a joyful mood (*n* = 1) were reported positive changes (Table 2).

**Table 2:** Behavioral changes after LEV application in dogs with pre-existing behavioral abnormalities (group 1).

**Behavioral change**	**Frequency of observed behavioral changes *n* = 63 (100%)**
Negative behavioral change	6 (10%)
Intensification of pre-existing behavioral abnormalities	41 (65%)
Positive behavioral change	16 (25%)

Of the 44 dogs, 4/44 dog owners reported negative changes in the behavior of their dogs after LEV treatment. Negative changes were stated as at least one additional behavioral factor in comparison to the behavioral status before treatment. Reported changes were anxiety (*n* = 2), aimless behavior (*n* = 2) and depression (*n* = 2) (Table 2). In the current study, depression was defined as agitation shown by the dog if disturbed from sleep and reduced interest in activities (23), adding reduced happiness as change of mental state. One of these four dogs developed an intensification of its newly reported negative behavior factor after an increase in dosage (14.4 mg/kg TID to 28.6 mg/kg TID). The onset of the behavioral changes became noticeable in all four dogs within the first 2 weeks of LEV treatment. Information on reversibility of the behavioral signs was only available on one dog, since two of the dogs were still being treated with LEV and information was not provided by the owner of one dog. Seizure frequency (*p* = 0.5), amount of administered AEDs (*p* = 0.84), age at onset of the first epileptic seizure (*p* = 0.23), and gender (*p* = 0.73) did not interrelate with a negative change in behavior.

14/44 (31.8%) dogs showed an intensification of their pre- existing behavior abnormalities under LEV therapy. These abnormalities included anxiety (*n* = 11), attention seeking behavior (*n* = 8), depression (*n* = 6), aimless behavior (*n* = 6), aggression (*n* = 6), hyperactivity (*n* = 3), and decreased learning ability (*n* = 1) (Table 2). In three of these 14 dogs their behavior abnormalities became more obvious after an increased dosage of LEV. 1/14 dogs developed aggressive behavior for the first time after increasing the dose (28.7 mg/kg TID to 37.5 mg/kg QID). Moreover, intensification of behavioral abnormalities occurred within the first 2 weeks in eight of the 14 dogs. Information on reversibility of the behavioral signs was only available on three dogs, since six of the dogs were still being treated with LEV, information was not provided by the owner of three dogs and two dogs were reported with an irreversible change in behavior. Seizure etiology of these two dogs were IE and StE. No statistical association could be detected for seizure frequency, behavioral abnormalities (*p* = 0.44), age at onset of the first epileptic seizure (*p* = 0.56), amount of administered AEDs (*p* = 0.45) or gender (*p* > 0.05).

16/44 dogs had no reported change or intensification in behavior during LEV treatment. However, five of these 16 dogs developed side effects like sedation and ataxia.

#### Dogs Without Behavioral Abnormalities Before LEV Treatment (Group 2; n = 40)

The dogs without a history of behavioral abnormalities had a median age of 5.75 years (range, 0.33–13 years) and a median weight of 19.75 kg (range, 3.5–68 kg). Dogs had either IE (*n* = 33), StE (*n* = 6), or RS (*n* = 1). Study participants of group two were six intact females, eight spayed females, 13 intact males and 13 neutered males. In 10 dogs LEV was given as monotherapy for a median duration of 1 month (range, 0.5–21 months). Nine of these ten dogs had a median dosage of 22.7 mg/kg TID (range, 13.9–26.3 mg/kg TID), while the remaining dog received a dosage of 17.2 mg/kg BID. In nine dogs LEV was applied as add-on treatment to other AEDs for a median time of 45 months (range, 1–75 months). In six of these nine dogs the median dosage was 25.7 mg/kg TID (range, 14.2–43.1 mg/kg TID).The remaining three dogs were treated with a median dosage of 38.5 mg/kg QID (range, 25–40 mg/kg QID). Moreover, 21 dogs were treated with LEV pulse therapy according to the schemes described in group one. Sixteen dogs were seizure free at the time of the study. Four of these 16 dogs were treated with long-term LEV monotherapy. Twenty-three dogs had still recurrent epileptic seizures and in one dog no information about seizure occurrence was available. All owners were asked to report seizure frequency and type of seizures of their dogs. A seizure frequency of at least one generalized seizure ≤4 weeks was reported in 25 dogs. Four dogs had epileptic seizures every 4–12 weeks. Three dogs had no pattern of seizure recurrence and in eight dogs no data about the seizure frequency were available. Thirty-eight dogs had generalized seizures, while two dogs showed focal seizures. Besides, 31 dogs were suffering from cluster seizures. In 17 dogs the first seizure occurred during their first 2 years of life, 23 dogs were older than 2 years at the time of first seizure occurrence. Moreover, all dogs in group two were treated with AEDs as permanent therapy. Twenty-four dogs were treated with one AED, eight dogs with two AEDs, seven dogs with three AEDs and one dog with four AEDs.

Of the 40 dogs, 5/40 dog owners graded the behavioral change after LEV treatment in a positive way. Reported changes were calmer mood (*n* = 3), increased activity (*n* = 1), and a joyful mood (*n* = 1) (Table 3).

**Table 3:** Behavioral changes after LEV application in dogs without behavioral abnormalities (group 2).

**Behavioral change**	**Frequency of observed behavioral changes *n* = 26 (100%)**
Negative behavioral change	21 (81%)
Positive behavioral change	5 (19%)

Of the 40 dogs, 10/40 dog owners reported negative behavioral changes for the first time after LEV treatment. Reported changes were anxiety (*n* = 6), depression (*n* = 4), aggression (*n* = 3), attention seeking behavior (*n* = 3), aimless behavior (*n* = 2), decreased learning ability (*n* = 2), hyperactivity (*n* = 1) (Table 3). In one of these ten dogs behavioral factors became more obvious after an increased dosage (19.7 mg/kg TID to 26.3 mg/kg TID). Behavioral changes occurred in 7/10 dogs during the first 2 weeks of LEV treatment. Statistical analysis revealed significance for the occurrence of negative behavioral effects within the first 2 weeks of treatment in this subgroup (*p* < 0.0001) and the whole study population (*p* = 0.0001). Information on reversibility of the behavioral signs was only available on five dogs, since three of the dogs were still being treated with LEV, information was not provided by the owner of one dog and one dog had an irreversible change in behavior after discontinuation of LEV. Seizure etiology was IE for the dog which had been reported with an irreversible change in behavior.

Negative behavioral changes were not associated with seizure frequency (*p* = 0.77), epilepsy etiology (*p* = 0.098), gender (*p* = 0.76), age at onset of the first epileptic seizure (*p* = 0.37) or the amount of administered AEDs (*p* = 0.83) within group two.

25/40 dogs developed no changes in their behavior during LEV treatment. Only two dogs had side effects like sedation and ataxia.

In the current study, 58 dogs were treated with phenobarbital, 15 dogs received imepitoin, 20 dogs potassium bromide, one dog was on gabapentin and 39 dogs had to take LEV as long-term antiseizure treatment. These AEDs were either administered alone or in combination. Additional information regarding study groups are published in supplementary material (Supplementary Table 1).

Furthermore, an association of age at onset of the first epileptic seizure to negative and positive behavioral changes after LEV administration was also statistically evaluated for all dogs diagnosed with IE to minimize a possible influence of other seizure etiologies as StE and RS on age at onset of the first seizure and therefore biasing the results. Analysis showed no association for occurring behavioral negative (*p* = 0.89) or positive (*p* = 0.87) changes for age at onset of the first epileptic seizure in dogs with IE. In addition, negative behavioral changes were not associated with seizure frequency (*p* = 0.34), gender (*p* = 0.63), or the amount of administered AEDs (*p* = 0.74) calculated for all dogs diagnosed with IE (*n* = 58) within this study population.

Due to the fact that no association could be found between the checked parameters except pre-existing behavioral abnormalities and SE, the authors conclude that the described changes in behavior are caused by LEV administration.

## Discussion

A bidirectional relation between epilepsy and behavioral problems is suspected in human medicine (26). People suffering depression or trying to commit suicide are at higher risk for developing unprovoked epileptic seizures (27). Moreover, the prevalence of major depression occurs more frequently in patients with epilepsy (28). Behavioral changes due to an epileptic cause (23, 29) and its impact on quality of life are also in veterinary medicine a topic of increased interest (30). In the current study, an association between behavioral changes and epilepsy could be detected within group one. However, a high percentage of dogs with StE (75%) were present in this group, when compared to the whole study population. It is known that behavioral changes in dogs with StE, especially in forebrain lesions, may occur and can be detected in neurological examination (31).

In human medicine the age at onset of the first epileptic seizure seems to have an influence on behavior. Children with epilepsy have an increased risk to develop behavioral abnormalities (32). This is in line with results of a veterinary study by Jokinen et al. (29). Jokinen et al. assume possible negative effects of the developing brain or the presence of prior neurobiological effects (29). In the current study with a heterogenous group of dogs, age of onset of the first epileptic seizure was not a risk factor for behavioral problems. However, different results may occur using other cutoff parameters.

Seizure frequency and its influence on behavior is subject of controversial discussion in human as well as in veterinary medicine (23, 29, 33, 34). In the current study we were not able to find an association between seizure frequency and behavioral changes.

An influence of sex on behavior in dogs with epilepsy is assumed (23, 29). For example, neutered dogs were reported to be more aggressive (29). In contrast Shihab and colleagues described an increased occurrence of abnormal perception, attachment disorder, and apathetic behavior in male dogs (23). No link between sex and behavioral changes could be found in the current study.

In addition, transient behavioral changes can occur in every state (prodromal phase, ictus, postictal phase) of an epileptic seizure (3, 35, 36). Due to potential altered postictal behavior and the fact that LEV was administered as pulse therapy (*n* = 45) directly after an epileptic seizure, transient behavioral changes could be mistaken as behavioral side effects of LEV. Therefore, the answers of the owners were verified at the end of the survey using validated questionnaires (22, 23). Using this double checking procedure and asking about long term changes, postictal changes were ruled out as best as possible.

Polypharmacy may also lead to behavioral changes (37). Effects of polypharmacy on behavior could not be detected in the current study. A possible explanation is the small number of dogs, which were treated with more than two AEDs (*n* = 14). However, the behavioral side effects of other AEDs than LEV should also be considered. Reported side effects for phenobarbital are aggression, restlessness and hyperactivity (20, 38–40). Imepitoin may also cause behavioral changes such as hyperactivity and aggression (41–44). Moreover, potassium bromide is also reported to cause these two behavioral changes (10). In addition, gabapentin may also influence behavior, as reported in children (45).

In the current study, 11% of the dogs were aggressive and 5% hyperactive after LEV treatment. The incidence of these two behavioral factors is comparable with other AEDs used in veterinary medicine (20, 38–44). A study in human medicine performed by White et al. (14) showed that 38 of the 553 patients were forced to LEV discontinuation due to behavioral side effects. Risk factors for discontinuation were fast titration rate to maximum dosage, StE and pre-existing behavioral problems (14, 46). In the current study, we were able to partly confirm these results in a dog model. 31.8% of the dogs with pre-existing behavioral abnormalities developed an intensifying of behavioral problems after LEV treatment. Comparing the dogs with an intensification of their behavior factors in relation to the whole study group, 16.7% of the 84 dogs showed an intensifying in their behavior.

Anxiety, depression and attention seeking behavior were the three most reported behavioral intensifications or negative behavioral changes in our study population. In total, 16.7 % of the dogs showed a worsening of their prior behavior after LEV treatment. Comparable results were found in human medicine (47).

There are diverse discussions on the influence of LEV dosage and occurrence of adverse effects in human medicine (14, 16, 48). In veterinary medicine, Packer et al. (25) described that dogs with LEV pulse therapy displayed significant more adverse effects than dogs under LEV maintenance treatment (~20 mg/kg TID). The most reported side effects within the pulse treatment group were ataxia, sedation, polyphagia and polydipsia (25). In the current study no clear association could be found between a high dosage and the occurrence of behavioral changes. However, within group 1, dogs are reported to develop more obvious behavioral changes in comparison to initial LEV treatment after an increase in LEV dosage. These observations indicate that an increase in dosage should be avoided in cases of negative adverse behavioral effects or an intensifying of prior behavioral problems after initial therapy.

Behavioral intensifications or negative behavioral changes became noticeable in 19 of the 28 dogs within the first 2 weeks after LEV treatment. Statistical analysis revealed significance for the occurrence of negative behavioral changes within the first 2 weeks of LEV treatment. This result is supported by research in human medicine (48), side effects occurred especially within the first 4 weeks of treatment (48).

Intensifications in behavior as well as negative behavioral changes after LEV treatment were reversible in 9 of the 28 dogs after discontinuation of LEV application. It can be assumed that negative behavioral changes are mostly reversible, considering research in human medicine (48) and our own results. The relatively low amount of reversibility in the current study can be explained with the fact that 11 of the 28 dogs were still treated with LEV at the time of data evaluation. Nevertheless, three dogs were reported to have irreversible behavioral changes after LEV treatment. One dog were diagnosed with StE, which might be a conclusive explanation. However, an appropriate explanation for the other two dogs diagnosed with IE is lacking. Severe brain damage due to seizures, misinterpretation by the dog owners or the drug itself could be the reason for irreversible change in behavior.

However, not only negative behavioral changes or intensifications of prior behavior factors can occur after LEV administration. Lagae et al. (49) reported positive behavioral changes in a study with epileptic children receiving LEV. Twenty-five percent of the children displayed positive behavioral changes or an improved alertness (49). This is supported by a veterinary study (5). Volk and colleagues reported that 57% of dog owners observed an increased liveliness and interactivity of their dogs (5). In the current study, comparable results were found. 17.9% of the dogs had an improvement of their behavior after LEV treatment as reported by the owners. Calmer behavior, increased activity and more energy were the three most reported positive behavioral changes in our study. Calmer behavior can result in a more stabile mood, however the sedative effect of LEV may have added to this effect. Interestingly, only five of the dogs for which were a positive behavioral change reported were free from seizures. Besides, in total study population 25 dogs were seizure free, indicating and further confirming that no association between seizure frequency and behavioral changes could be detected in this study.

Limitations of this study were the inclusion of dogs with various etiologies of seizures into a single group. As behavioral changes can be a manifestation of the disease associated with StE, RS, and IE the study findings might have been influenced by the underlying cause for epileptic seizures. However, this influence was minimized by evaluating the dogs' behavior before and after LEV administration. Furthermore, the questionnaire included validated standardized questions concerning dogs' behavior and all answers were rechecked with the medical records including the results of the neurological examination. The questions were originally validated by behavioral specialists (22–24). The study relied on the owners' retrospective recall which might have led to a certain source of bias. However, we did our best to minimize the bias, e.g., by evaluating the influence of epileptic seizure frequency, sex, epilepsy etiology, age at onset of the first epileptic seizure and polypharmacy on occurrence of behavioral changes after LEV application and by several double checkings as described in material and methods.

In conclusion, positive and negative behavioral changes under LEV treatment occur in dogs.

In total 43 (51%) of 84 examined dogs developed a change or an intensifying of their behavior after LEV treatment.

Pre-existing behavioral problems should be taken into account, when deciding for LEV treatment and potential intensifying or change in the dogs' behavior should be discussed with the owners. Besides, an increase in dosage should be avoided in cases of negative behavioral effects or an intensifying of prior behavioral problems after initial therapy, since further changes could occur. In the current study, negative behavioral changes appeared mostly within the first 2 weeks of treatment. However, changes may be reversible and severe negative behavior changes leading to drug discontinuation were rarely observed. In addition, improvement of behavior factors is also possible and can be favorable for the treated dogs.

## Data Availability Statement

The datasets generated for this study are available on request to the corresponding author.

## Ethics Statement

The study was conducted in accordance with the German Animal Welfare Act within the law of animal welfare, following the ethical guidelines of the University of Veterinary Medicine Hannover (approval of the thesis commission).

## Author Contributions

AT and JE were responsible for the conception of the study. Data acquisition was done by JN, FR, EH, and JE. Statistical analysis, data analysis, and manuscript writing was performed by JE. KR provided statistical advice. AT supervised data collection and manuscript editing.

### Conflict of Interest

The authors declare that the research was conducted in the absence of any commercial or financial relationships that could be construed as a potential conflict of interest.

## Abbreviations

LEV: Levetiracetam
AED: antiepileptic drug
StE: structural epilepsy
IE: idiopathic epilepsy
RS: reactive seizures
BID: twice daily
TID: three times daily
TIER 1, 2, 3: confidence level for the diagnosis of IE
QID: four times daily.
