# Supplementary material for: Behavioral Changes Under Levetiracetam Treatment in Dogs
Source: Front Vet Sci. 2020 Apr 3;7:169. doi: 10.3389/fvets.2020.00169 (PMC7146871; doi:10.3389/fvets.2020.00169)
Supplement: Supplementary file 1 [file Table_1.DOCX]

Supplementary Material

# Supplementary Data


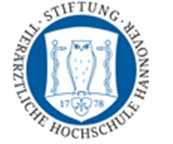
**Verhaltensänderungen bei epileptischen Hunden durch Levetiracetamgabe**

Sehr geehrte Damen und Herren,

mein Name ist Johannes Erath.

Ich bin Doktorand in der Klinik für Kleintiere der Stiftung Tierärztliche Hochschule Hannover, in der Abteilung Neurologie, unter der Leitung von Frau Prof. Dr. Tipold.

Ich beschäftige mich mit einer Studie zum Antiepileptikum Levetiracetam bei epileptischen Hunden.

Levetiracetam ist ein potentes Antiepileptikum und reduziert signifikant die Anfallshäufigkeit. Dabei verfügt es über wenige systemische Nebenwirkungen und wird generell sehr gut vertragen. In Einzelfällen konnten jedoch Verhaltensänderungen erfasst werden.

Meine Studie wird sich mit diesem Aspekt beschäftigen.

Hierfür benötige ich nun Ihre Hilfe, denn nur Sie können das Verhalten Ihres Tieres am Besten beurteilen.

Mit Ihrer Mitarbeit tragen Sie dazu bei, dass wir Ihr Tier und folgende Patienten noch effektiver therapieren können. Außerdem sind Sie uns eine große Hilfe dabei, die Erkrankung Epilepsie, sowie, die daraus resultierenden Verhaltensänderungen besser zu verstehen.

Ich danke Ihnen sehr für Ihre Teilnahme und möchte Sie ermuntern, gemeinsam mit uns, an neuen Therapieoptionen zu arbeiten.

Sollten Sie Fragen zu dieser Studie haben, können Sie mich sehr gerne unter folgender Telefonnummer oder E-Mail kontaktieren:

Telefonnummer: - oder- (Sekretariat der Klinik)

E-Mail Adresse: -

Bitte beachten Sie, dass die Auswertung Ihrer Angaben im Einklang mit der Datenschutz-Grundverordnung (DSGVO) stattfinden wird. Alle Daten werden sicher und vertraulich verarbeitet.

Die Umfrage wurde mit Hilfe einer hierfür vorgesehenen Software von LimeSurvey auf dem Klinik-Server entworfen. Darüber hinaus kann die Datenschutzrichtlinie von LimeSurvey online eingesehen werden: https://www.limesurvey.org/de/richtlinien/datenschutzrichtlinie ;

diese ist ebenfalls Gegenstand dieser Erklärung.

Sie haben das Recht auf Auskunft, Berichtigung, Löschung, Einschränkung der Datenübertragbarkeit, Einschränkung der Weiterleitung Ihrer Daten an Dritte, Widerruf und Widerspruch im Sinne der DSGVO.

Durch das Ausfüllen dieser Umfrage stimmen Sie zu, dass die Informationen durch die Kleintierklinik der Stiftung Tierärztliche Hochschule Hannover verarbeitet werden können. Dies beinhaltet auch die Nutzung zu Lehr-, Forschungs-, Vortrags- und Publikationszwecken.

Wenn Sie glauben, dass die Verarbeitung Ihrer Daten gegen das Datenschutzrecht verstößt oder Ihre datenschutzrechtlichen Ansprüche in einer anderen Weise verletzt worden sind, wenden Sie sich gern an mich:

Johannes Erath Doktorand Neurologie (Telefonummer/Mailadresse)

**Sind Sie mit der Datenschutzerklärung einverstanden?**

- Ja
- Nein

**Beginn der Umfrage:**

1. Um welche Rasse handelt es sich bei Ihrem Hund?

___________________________________________________________________

1. Welches Geschlecht hat Ihr Hund?

- Männlich
- Männlich-kastriert
- Weiblich
- Weiblich-kastriert

1. Was wiegt Ihr Hund?

(Angabe in **kg**)

___________________________________________________________________

1. Wie alt ist Ihr Hund?

(Angabe in **Jahren**)

___________________________________________________________________

1. Seit wann leidet Ihr Hund an Epilepsie?

(Wenn möglich Angabe des Datums des ersten Anfalles)

___________________________________________________________________

1. Leidet Ihr Hund noch an epileptischen Anfällen?

- Ja
- Nein
- Wenn Nein,
- Seit wann ist Ihr Hund anfallsfrei?

(Wenn möglich Angabe des Datums)

________________________________________________

1. Ist die Ursache für diese Erkrankung bei Ihrem Hund bekannt?

- Ja
- Nein
- Wenn Ja,
- Ursache der Erkrankung

  ________________________________________________

1. Steht Ihr Hund unter einer antiepileptischen Dauertherapie?

- Ja
- Nein
- Wenn Ja,
- Welches Medikament/ Welche Medikamente geben Sie?

(Angabe des Präparatenamens und/oder des Wirkstoffes)

________________________________________________

1. Wie ist die Dosierung der jeweiligen Medikamente?

- Angabe der Häufigkeit der täglichen Gabe sowie der Tablettenmenge und –Dosierung
- Bsp: 2xtgl 1 Tablette Levetiracetam 250mg

Ihre Angabe:

___________________________________________________________________

1. In welchen Zeitabständen ereignen sich die Anfälle?

(Bitte wählen Sie nur **eine** der folgenden Antworten aus.)

- täglich
- im 1-2 Wochen Abstand
- alle 3-4 Wochen
- andere Zeitabstände:

  ____________________________________________________________

1. Wie lange dauert ungefähr der Krampfanfall?

(Bitte wählen Sie nur **eine** der folgenden Antworten aus.)

- wenige Sekunden
- 1-2 Minuten
- 3-5 Minuten
- 6-10 Minuten
- länger

1. Wenn Ihr Hund Krampfanfälle hat, kommen mehrere Anfälle hintereinander vor (Cluster)?

- Ja
- Nein, nur ein Anfall pro 24 Stunden
- Wenn ja,

Wie häufig ereignen sich die Krampfanfälle in 24 Stunden?

(Bitte wählen Sie nur **eine** der folgenden Antworten aus.)

- 2 zeitlich getrennte Anfälle pro 24 Stunden
- 3-5 zeitlich getrennte Krampfanfälle pro 24 Stunden
- mehr als 5 Krampfanfälle pro 24 Stunden

1. Wie sehen diese Krampfanfälle aus?

(Bitte wählen Sie **alle** zutreffenden Antworten aus.)

- Tier fällt um und ist in Seitenlage
- Tier war ansprechbar
- Tier war nicht ansprechbar
- Tier macht mit allen 4 Beinen Ruderbewegungen
- unkontrollierter Urin-und Kotabsatz
- Speicheln
- abnormale Augenbewegungen
- Tier wird am ganzen Körper steif
- Krampf beginnt an einer Stelle und breitet sich auf den ganzen Körper aus

1. Sind die Krampfanfälle auf einen bestimmten Körperteil begrenzt?

- Ja
- Nein
- Wenn ja,
- welcher Körperteil ist betroffen?

  ________________________________________________

1. Sind abgesehen von der Epilepsie weitere Erkrankungen bei Ihrem Hund bekannt?

- Ja
- Nein

- Wenn ja,
- welche zusätzlichen Erkrankungen liegen vor?

  ________________________________________________

1. Hat Ihr Hund Verhaltensauffälligkeiten entwickelt, seitdem er Krampfanfälle hat?
   (Das heißt: Stehen die Verhaltensauffälligkeiten in einem **zeitlichen Zusammenhang mit dem Beginn der Krampfanfälle**?)

- Ja
- Nein
- Wenn Ja,

Wie äußern sich diese Verhaltensauffälligkeiten?
(Bitte benennen Sie hier nur Auffälligkeiten, die **VOR** Levetiracetamgabe von Ihnen bemerkt wurden. Bitte wählen Sie **alle** zutreffenden Antworten aus.)

- - ist leicht reizbar
  - mag keine fremden Hunde
  - mag keine fremden Menschen
  - lernt langsam
  - ängstlich
  - neigt zu Aggressivität
  - ist leicht abzulenken
  - ist überaus lebhaft
  - unerklärliches Verhalten wie plötzliches Bellen, Schatten jagen oder in die Luft starren vermutlich durch Halluzinationen
  - sucht die Aufmerksamkeit des Besitzers oder übriger Personen
  - vermindertes Interesse an Aktivitäten
  - wirkt weniger freudig
  - Hund wird aggressiv, wenn er beim Schlafen gestört wird
  - Keine der oben genannten, sondern:

    ________________________________________________

1. Würden Sie sagen, dass sich diese Verhaltensauffälligkeiten nach Levetiracetamgabe **verstärkt** haben?

(Frage muss nur beantwortet werden, wenn Sie **Frage 16** mit **Ja** beantwortet haben.)

- Ja
- Nein
- Wenn Ja,

Welche der folgenden Verhaltensauffälligkeiten haben sich verstärkt seitdem Ihr Hund unter Levetiracetamtherapie steht?

(Bitte wählen Sie **alle** zutreffenden Antworten aus.)

- ist leichter reizbar
- Abneigung gegenüber fremden Hunden hat zugenommen
- Abneigung gegenüber fremden Menschen hat zugenommen
- lernt langsamer
- ist ängstlicher
- vermehrt aggressiv
- ist leichter abzulenken
- ist äußerst lebhaft
- vermehrtes unerklärliches Verhalten wie plötzliches Bellen, Schatten jagen oder in die Luft starren
- sucht vermehrt die Aufmerksamkeit des Besitzers oder übriger Personen
- verstärkt desinteressiert an Aktivitäten
- wirkt weniger freudig
- wird aggressiv, wenn er beim Schlafen gestört wird
- keine der oben genannten, sondern:

  ________________________________________________

1. Weshalb bekam Ihr Hund Levetiracetam verschrieben?

(Bitte wählen Sie **alle** zutreffenden Antworten aus.)

- aufgrund mangelnder Wirksamkeit der anderen Antiepileptika
- aufgrund der hohen Anfallsfrequenz
- da er 2 oder mehr zeitlich voneinander getrennte Anfälle pro 24 Stunden hatte (Cluster)
- mein Hund leidet an Leberproblemen
- Sonstiges:

  ____________________________________________________________

1. In welcher Dosierung erhält Ihr Hund Levetiracetam?

Wie oft geben Sie die Tabletten täglich?

(Angabe nur in **Zahlen**)

___________________________________________________________________

Wie viele Tabletten geben Sie pro Gabe?

(Angabe nur in **Zahlen**)

___________________________________________________________________

Wie hoch ist die Dosierung der einzelnen Tabletten (in mg)?

(mögliche Antworten: 250 mg, 500mg, 750 mg, 1000 mg)

___________________________________________________________________

1. Wurde die Dosierung von Levetiracetam erhöht?

- Ja
- Nein
- Wenn Ja,

geben Sie bitte das Zeitintervall sowie die Tablettenmenge und –dosierung an

- Beispiel:
  nach 7 Tagen Erhöhung von Levetiracetam von 2x täglich 1 Tablette Levetiracetam 250 mg auf 2x täglich 1,5 Tabletten Levetiracetam 250 mg

  ____________________________________________________________

1. Zeigte Ihr Hund im Zuge der Dosiserhöhung von Levetiracetam Verhaltensänderungen?

(Frage muss nur beantwortet werden, wenn Sie **Frage 20** mit **Ja** beantwortet haben)

- Ja
- Nein
- Wenn ja,

Wie äußerten sich diese Verhaltensauffälligkeiten?

(Bitte wählen Sie **alle** zutreffenden Antworten aus.)

- ist leicht reizbar
- mag keine fremden Hunde
- mag keine fremden Menschen
- lernt langsam
- ängstlich
- neigt zu Aggressivität
- ist leicht abzulenken
- ist überaus lebhaft
- unerklärliches Verhalten wie plötzliches Bellen, Schatten jagen oder in die Luft starren, vermutlich durch Halluzinationen
- sucht die Aufmerksamkeit des Besitzers oder übriger Personen
- vermindertes Interesse an Aktivitäten
- wirkt weniger freudig
- Hund wird aggressiv, wenn er beim Schlafen gestört wird
- Keine der oben genannten, sondern:
  ________________________________________________

1. Zeigte ihr Hund **NACH** Levetiracetamgabe Verhaltensänderungen?

- Ja
- Nein
- Wenn Ja,

Wie äußerten sich diese Verhaltensänderungen?

(Bitte wählen Sie **alle** zutreffenden Antworten aus.)

- ist leicht reizbar
- mag keine fremden Hunde
- mag keine fremden Menschen
- lernt langsam
- ängstlich
- neigt zu Aggressivität
- ist leicht abzulenken
- ist überaus lebhaft
- unerklärliches Verhalten wie plötzliches Bellen, Schatten jagen oder in die Luft starren, vermutlich durch Halluzinationen
- sucht die Aufmerksamkeit des Besitzers oder übriger Personen
- vermindertes Interesse an Aktivitäten
- wirkt weniger freudig
- Hund wird aggressiv, wenn er beim Schlafen gestört wird
- keine der oben genannten, sondern:

  ________________________________________________

1. Wann traten die Verhaltensänderungen nach Gabe von Levetiracetam auf?

(Frage muss nur beantwortet werden, wenn Sie **Frage 22** mit **Ja** beantwortet haben)

(Bitte wählen Sie nur **eine** der folgenden Antworten aus.)

- direkt nach der ersten Gabe von Levetiracetam
- nach1-2 Wochen nach der ersten Gabe von Levetiracetam
- nach 3-4 Wochen nach der ersten Gabe von Levetiracetam
- anderer Zeitabstand:

  ____________________________________________________________

1. Verschwanden die Verhaltensänderungen nach Absetzen von Levetiracetam?

(Frage muss nur beantwortet werden, wenn Sie **Frage 22** mit **Ja** beantwortet haben)

- Ja
- Nein
- Levetiracetam wurde nicht abgesetzt
- Sonstiges:

  ____________________________________________________________

1. Hatte die Levetiracetamgabe einen positiven Effekt auf das Verhalten Ihres Tieres?

- Ja
- Nein
- Wenn Ja,

(Bitte wählen Sie **alle** zutreffenden Antworten aus.)

- gesteigerte Aktivität
- mehr Energie
- ruhiger
- gehorsamer
- andere positive Effekte:

  ________________________________________________

1. Ist Ihr Hund nach der Gabe von Levetiracetam anfallsfrei geworden?

- Ja
- Nein, aber verminderte Anfallsfrequenz
- Keine Wirkung
- Sonstiges:

  ____________________________________________________________

1. Nach welcher Zeitspanne wurde Ihr Hund anfallsfrei?

(Frage muss nur beantwortet werden, wenn Sie **Frage 26** mit **Ja** beantwortet haben)

(Bitte wählen Sie nur **eine** der folgenden Antworten aus.)

- direkt nach der ersten Gabe von Levetiracetam
- nach 1-2 Wochen nach der ersten Gabe von Levetiracetam
- nach 3-4 Wochen nach der ersten Gabe von Levetiracetam
- Sonstiges:

  ____________________________________________________________

1. Bekam Ihr Hund jemals eine Levetiracetamimpulstherapie ?

(Dabei handelt es sich um eine ca. einwöchige Therapie, um die Clusteranfälle einzudämmen.)

- Ja
- Nein
- Weiß ich nicht

1. Zeigte Ihr Hund unter der Levetiracetamimpulstherapie Verhaltensänderungen?

(Frage muss nur beantwortet werden, wenn Sie **Frage 28** mit **Ja** beantwortet haben)

- Ja
- Nein
- Wenn ja,

(Bitte wählen Sie **alle** zutreffenden Antworten aus)

- ist leicht reizbar
- mag keine fremden Hunde
- mag keine fremden Menschen
- lernt langsam
- ängstlich
- neigt zu Aggressivität
- ist leicht abzulenken
- ist überaus lebhaft
- unerklärliches Verhalten wie plötzliches Bellen, Schatten jagen oder in die Luft starren vermutlich durch Halluzinationen
- sucht die Aufmerksamkeit des Besitzers oder übriger Personen
- vermindertes Interesse an Aktivitäten
- wirkt weniger freudig,
- Hund wird aggressiv, wenn er beim Schlafen gestört wird
- Keine der oben genannten, sondern:

  ________________________________________________

**Evaluation einiger Verhaltensparameter VOR Levetiracetamgabe**

Beantworten Sie diesen Abschnitt bitte im Hinblick darauf wie Ihr Hund sich Ihnen **VOR** Levetiracetamgabe präsentiert hat.

1. Hund reagiert ängstlich oder besorgt,

(Bitte wählen Sie **alle** zutreffenden Antworten aus.)

- wenn sich ihm unbekannte Hunde nähern
- wenn er sich in neuer oder unbekannter Umgebung befindet
- wenn eine unbekannte Person das Haus betritt
- wenn unbekannte Personen zu Gast sind
- wenn sich plötzliche oder, für den Hund unvorhersehbare, Bewegungen ereignen
- in Verbindung mit plötzlichen oder lauten Geräuschen
- während eines Gewitters
- wenn er unbekannten Situationen ausgesetzt wird
- wenn sich ihm unbekannte Personen außerhalb des Hauses nähern
- in Verbindung mit Wind oder im Wind wehenden Objekten
- wenn er allein gelassen wird im Haus oder wenn eine ihm vertraute Person das Haus verlässt

1. Hund reagiert aggressiv,

(Bitte wählen Sie **alle** zutreffenden Antworten aus.)

- wenn sich ihm ein unbekannter Hund nähert, während des Spazierengehens oder während er an der Leine geführt wird
- auf unbekannte Hunde, wenn diese zu Besuch sind
- wenn der Hund berührt bzw. manipuliert wird
- wenn Menschen oder Hunde am Haus vorbeigehen
- wenn sich Passanten dem Hund nähern während des Spazierganges
- wenn Essen, Knochen oder Spielzeug entfernt wird
- wenn er zurechtgewiesen oder bestraft wird
- wenn man sich ihm nähert während er frisst
- wenn eine ihm bekannte Person das Haus betritt
- wenn ein Fremder das Haus betritt

1. Hat Ihr Hund jemals versucht

(Bitte wählen Sie **alle** zutreffenden Antworten aus.)

- andere Hunde zu beißen
- unbekannte Personen zu beißen

1. Hund wird beobachtet,

(Bitte wählen Sie **alle** zutreffenden Antworten aus.)

- bellend ohne ersichtlichen Grund
- Lichtpunkte oder Schatten jagend
- ziellos umherwandernd oder auf und abgehend
- in die Luft starrend

1. Hund,

(Bitte wählen Sie **alle** zutreffenden Antworten aus.)

- zeigt Erregung, wenn er beim Schlafen gestört wird
- zeigt vermindertes Interesse an Aktivitäten
- wirkt weniger freudig

1. Hund,

(Bitte wählen Sie **alle** zutreffenden Antworten aus.)

- zeigt enge Bindung zu einem bestimmten Familienmitglied
- neigt dazu einem bestimmten Familienmitglied von Raum zu Raum zu folgen
- neigt dazu sich nahe oder in direktem Kontakt zu einem Familienmitglied aufzuhalten
- neigt dazu ein Familienmitglied/andere Person anzustupsen oder mit der Pfote zu berühren, um dessen Aufmerksamkeit zu gewinnen
- reagiert Aufmerksamkeit fordernd, wenn ein Familienmitglied/andere Person Zuneigung gegenüber einer anderen Person oder Tier zeigt

1. Hund,

(Bitte wählen Sie **alle** zutreffenden Antworten aus.)

- kommt direkt zurück, wenn er gerufen wird
- macht direkt Sitz, wenn es ihm befohlen wird
- bleibt an Ort und Stelle, wenn es ihm befohlen wird
- bringt Stöckchen, Bälle oder andere Gegenstände
- wirkt so als ob er genau zuhört bei allem was der Besitzer sagt oder tut
- reagiert langsam auf Zurechtweisungen oder Bestrafungen
- braucht länger um neue Tricks und Aufgaben zu lernen

1. Hund,

(Bitte wählen Sie **alle** zutreffenden Antworten aus.)

- ist leicht abzulenken durch interessante Gerüche
- ist leicht abzulenken durch interessante Geräusche
- ist leicht abzulenken durch visuelle Effekte

1. Hund ist aufgeregt oder reagiert über,

(Bitte wählen Sie **alle** zutreffenden Antworten aus.)

- wenn ein Familienmitglied nach Hause kommt nach kurzer Abwesenheit
- wenn er mit einem Familienmitglied spielt
- wenn die Türklingel läutet
- kurz vor einem Spaziergang
- kurz vor einer Autofahrt
- wenn Besucher ankommen

**Evaluation einiger Verhaltensparameter NACH Levetiracetamgabe**

Beantworten Sie diesen Abschnitt bitte im Hinblick darauf wie Ihr Hund sich Ihnen **NACH** Levetiracetamgabe präsentiert hat.

1. Hund reagiert ängstlich oder besorgt,

(Bitte wählen Sie **alle** zutreffenden Antworten aus.)

- wenn sich ihm unbekannte Hunde nähern
- wenn er sich in neuer oder unbekannter Umgebung befindet
- wenn eine unbekannte Person das Haus betritt
- wenn unbekannte Personen zu Gast sind
- wenn sich plötzliche oder, für den Hund unvorhersehbare, Bewegungen ereignen
- in Verbindung mit plötzlichen oder lauten Geräuschen
- während eines Gewitters
- wenn er unbekannten Situationen ausgesetzt wird
- wenn sich ihm unbekannte Personen außerhalb des Hauses nähern
- in Verbindung mit Wind oder im Wind wehenden Objekten
- wenn er allein gelassen wird im Haus oder wenn eine ihm vertraute Person das Haus verlässt

1. Hund reagiert aggressiv,

(Bitte wählen Sie **alle** zutreffenden Antworten aus.)

- wenn sich ihm ein unbekannter Hund nähert, während des Spazierengehens oder während er an der Leine geführt wird
- auf unbekannte Hunde, wenn diese zu Besuch sind
- wenn der Hund berührt bzw. manipuliert wird
- wenn Menschen oder Hunde am Haus vorbeigehen
- wenn sich Passanten dem Hund nähern während des Spazierganges
- wenn Essen, Knochen oder Spielzeug entfernt wird
- wenn er zurechtgewiesen oder bestraft wird
- wenn man sich ihm nähert während er frisst
- wenn eine ihm bekannte Person das Haus betritt
- wenn ein Fremder das Haus betritt

1. Hat Ihr Hund jemals versucht,

(Bitte wählen Sie **alle** zutreffenden Antworten aus.)

- andere Hunde zu beißen
- unbekannte Personen zu beißen

1. Hund wird beobachtet,

(Bitte wählen Sie **alle** zutreffenden Antworten aus.)

- bellend ohne ersichtlichen Grund
- Lichtpunkte oder Schatten jagend
- ziellos umherwandernd oder auf und abgehend
- in die Luft starrend

1. Hund,

(Bitte wählen Sie **alle** zutreffenden Antworten aus.)

- zeigt Erregung, wenn er beim Schlafen gestört wird
- zeigt vermindertes Interesse an Aktivitäten
- wirkt weniger freudig

1. Hund,

(Bitte wählen Sie **alle** zutreffenden Antworten aus.)

- zeigt enge Bindung zu einem bestimmten Familienmitglied
- neigt dazu einem bestimmten Familienmitglied von Raum zu Raum zu folgen
- neigt dazu sich nahe oder in direktem Kontakt zu einem Familienmitglied aufzuhalten
- neigt dazu ein Familienmitglied/andere Person anzustupsen oder mit der Pfote zu berühren, um dessen Aufmerksamkeit zu gewinnen
- reagiert Aufmerksamkeit fordernd, wenn ein Familienmitglied/andere Person Zuneigung gegenüber einer anderen Person oder Tier zeigt

1. Hund,

(Bitte wählen Sie **alle** zutreffenden Antworten aus.)

- kommt direkt zurück, wenn er gerufen wird
- macht direkt Sitz, wenn es ihm befohlen wird
- bleibt an Ort und Stelle, wenn es ihm befohlen wird
- bringt Stöckchen, Bälle oder andere Gegenstände
- wirkt so als ob er genau zuhört bei allem was der Besitzer sagt oder tut
- reagiert langsam auf Zurechtweisungen oder Bestrafungen
- braucht länger um neue Tricks und Aufgaben zu lernen

1. Hund,

(Bitte wählen Sie **alle** zutreffenden Antworten aus.)

- ist leicht abzulenken durch interessante Gerüche
- ist leicht abzulenken durch interessante Geräusche
- ist leicht abzulenken durch visuelle Effekte

1. Hund ist aufgeregt oder reagiert über,

(Bitte wählen Sie **alle** zutreffenden Antworten aus.)

- wenn ein Familienmitglied nach Hause kommt nach kurzer Abwesenheit
- wenn er mit einem Familienmitglied spielt
- wenn die Türklingel läutet
- kurz vor einem Spaziergang
- kurz vor einer Autofahrt
- wenn Besucher ankommen

**Supplementary Figure 1:** Questionnaire of the study
