# Supplementary material for: Behavioral Changes Under Levetiracetam Treatment in Dogs
Source: Front Vet Sci. 2020 Apr 3;7:169. doi: 10.3389/fvets.2020.00169 (PMC7146871; doi:10.3389/fvets.2020.00169)
Supplement: Supplementary file 3 [file Data_Sheet_2.docx]

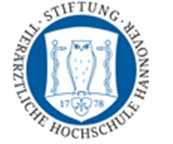
Supplementary Material

# Supplementary Data

**Behavioral changes under levetiracetam treatment in dogs**

Dear Sir or Madam,

my name is Johannes Erath.

I am a doctoral student at the in the Clinic for Small Animals at the University of Veterinary Medicine Hannover under supervision of Prof. Tipold.

I am studying the antiepileptic drug levetiracetam in epileptic dogs.

Levetiracetam is a potent antiepileptic drug which significantly reduces seizure frequency. Only mild to moderate side effects are reported for this drug and it is well tolerated. In some cases, behavioral changes after levetiracetam administration were detected.

My study will deal with this aspect.

Therefore, I need your help because you can evaluate your pet’s behavior best.

With your assistance, you can help us to treat following patients more effective. Additionally, you can significantly contribute to the comprehension of the disease epilepsy and the resulting behavioral changes.

I am thankful for your participation and I would like to encourage you to develop new therapeutic options together with us.

If there are any questions regarding this study, you can reach me via e-mail or phone.

Please note that the interpretation of our data is in accordance with General Data Protection Regulation. All data will be treated securely and confidentially.

The survey was done with help of a software called LimeSurvey and was designed at the server of the clinic. Additionally, it is possible to consult the Data Protection Directive of LimeSurvey online:

https://www.limesurvey.org/de/richtlinien/datenschutzrichtlinie;

This is also subject of this statement.

You have the right to information, correction, deletion, restriction of data handling, restriction of forwarding your data to third parties, revocation and objection in terms of the General Data Protection Regulation.

By completing this survey you accept that all given information can be handled by the Clinic for Small Animals at the University of Veterinary Medicine Hannover . This includes utilization for teaching, research, presentation and publication purposes.

If you believe that the procession of your data violates the data protection law or that your data protection rights have been violated in any other way, please contact me.

Johannes Erath doctoral student neurology (phone/ e-mail)

**Do you agree with the privacy statement?**

- Yes
- No

**Begin of the survey:**

1. Which breed is your dog?

___________________________________________________________________

1. Which gender is your dog?

- Intact male
- Neutered male
- Intact female
- Spayed female

1. What is your dog’s weight?

(specification in **kg**)

___________________________________________________________________

1. How old is your dog?

(specification in **years**)

___________________________________________________________________

1. Since when is your dog suffering from epilepsy?

(if possible, write the date of its first seizure)

___________________________________________________________________

1. Does your dog still suffer from epileptic seizures?

- Yes
- No
- If no,
- Since when is your dog free of seizures?

(if possible, write the exact date)

________________________________________________

1. Is the etiology of the seizures known?

- Yes
- No
- If yes,
- Seizure etiology

  ________________________________________________

1. Is your dog under permanent antiepileptic therapy?

- Yes
- No
- If yes,
- Which drug/ drugs?

(specification of the pharmaceutical preparation and/or substance)

________________________________________________

1. What is the dosage of the respective drugs?

- Specification of daily frequency of drug intake, the amount of tablets and its dosage
- e.g. twice daily 1 tablet levetiracetam 250mg

Your specification:

___________________________________________________________________

1. At what intervals do the seizures occur?

(Please choose only **one** of the following answers.)

- daily
- every 1-2 weeks
- every 3-4 weeks
- other:

  ____________________________________________________________

1. How long does a seizure of your dog last?

(Please choose only **one** of the following answers.)

- a few seconds
- 1-2 minutes
- 3-5 minutes
- 6-10 minutes
- longer

1. If your dog is seizuring, does it have multiple seizures (cluster seizures)?

- Yes
- No, only one seizure within 24 hours
- If yes,

How many seizures does your dog have within 24 hours?

(Please choose only **one** of the following answers)

- 2 seizures separated in time per 24 hours
- 3-5 seizures separated in time per 24 hours
- More than 5 separated seizures within 24 hours

1. How do the seizures look like?

(Please choose **every** suitable answer.)

- Dog is in lateral recumbancy
- Dog is responsive
- Dog is not responsive
- Dog paddles with every four legs
- Dog urinates/defactes uncontrolled
- Dog salivates
- Dog shows abnormal eye movement
- Dog becomes stiff all over the body
- Seizure starts on one part of the body and is then spreading all over the body

1. Are the seizures limited to a specific part of the body?

- Yes
- No
- If yes,
- Which part of the body?

  ________________________________________________

1. Does your dog have other diseases than epilepsy?

- Yes
- No

- If yes,
- Which other diseases?

  ________________________________________________

1. Has your dog developed behavioral abnormalities since its first seizure?

(this means: Are the behavioral abnormalities **in a chronologically context with the beginning of the seizures**?)

- Yes
- No
- If yes,

How do these behavioral abnormalities look like?
(Please only name behavioral abnormalities **BEFORE** levetiracetam administration. (Please choose **every** suitable answer.)

- - is easily irritable
  - does not like unfamiliar dogs
  - does not like unfamiliar persons
  - learns slowly
  - anxious
  - aggressive
  - is easily distracted
  - hyperactive
  - abnormal behavior as barking without any apparent cause, chasing shadows or staring into space, probably caused by halucinations
  - shows attention seeking behavior towards its owner or other persons
  - decreased interest in activities
  - depressive
  - is agitated if disturbed from sleep
  - none of these, but:

    ________________________________________________

1. Have these behavioral abnormalities **increased** since levetiracetam administration?

(Question only has to be answered, if you chose **yes** at **question 16**.)

- Yes
- No
- If yes,

Which of the following behavioral abnormalities have increased since levetiracetam intake?

(Please choose **every** suitable answer.)

- - is easily irritable
  - does not like unfamiliar dogs
  - does not like unfamiliar persons
  - learns slowly
  - anxious
  - aggressive
  - is easily distracted
  - hyperactive
  - abnormal behavior as barking without any apparent cause, chasing shadows or staring into space, probably caused by halucinations
  - shows attention seeking behavior towards its owner or other persons
  - decreased interest in activities
  - depressive
  - is agitated if disturbed from sleep
- none of these, but:

  ________________________________________________

1. What was the reason for levetiracetam adminstration?

(Please choose **every** suitable answer.)

- Inappropriate effectiveness of the other antiepileptic drugs
- High seizure frequency
- Dog had more than 2 seizures separated in time within 24 hours (cluster seizures)
- Dog has liver problems
- others:

  ____________________________________________________________

1. What is the levetiracetam dosage of your dog?

How many times per day do you give the tablets?

(Specification in **numbers**)

___________________________________________________________________

How many tablets do you give per administration?

(Specification in **numbers**)

___________________________________________________________________

What is the dosage of the tablets (in mg)?

(possible answers: 250 mg, 500 mg, 750 mg, 1000 mg)

___________________________________________________________________

1. Was the dosage of levetiracetam increased?

- Yes
- No
- If yes,

please write down time period, amount and dosage of tablets

e.g.:
increase of levetiracetam dosage from 1 tablet 250 mg twice daily to 1.5 tablets 250 mg twice daily after 7 days

____________________________________________________________

1. Did your dog have behavioral abnormalities after increasing the levetiracetam dosage?

(Question has only to be answered, if you chose **yes** at **question 20**.)

- Yes
- No
- If yes,

How did the behavioral abnormalities look like in your dog?

(Please choose **every** suitable answer.)

- - is easily irritable
  - does not like unfamiliar dogs
  - does not like unfamiliar persons
  - learns slowly
  - anxious
  - aggressive
  - is easily distracted
  - hyperactive
  - abnormal behavior as barking without any apparent cause, chasing shadows or staring into space, probably caused by halucinations
  - shows attention seeking behavior towards its owner or other persons
  - decreased interest in activities
  - depressive
  - is agitated if disturbed from sleep
  - None of these, but:

________________________________________________

1. Did your dog had behavioral abnormalities **AFTER** levetiracetam administration?

- Yes
- No
- If yes,

How did the behavioral abnormalities look like in your dog?

(Please choose **every** suitable answer.)

- - is easily irritable
  - does not like unfamiliar dogs
  - does not like unfamiliar persons
  - learns slowly
  - anxious
  - aggressive
  - is easily distracted
  - hyperactive
  - abnormal behavior as barking without any apparent cause, chasing shadows or staring into space, probably caused by halucinations
  - shows attention seeking behavior towards its owner or other persons
  - decreased interest in activities
  - depressive
  - is agitated if disturbed from sleep
  - None of these, but:

________________________________________________

1. When did your dog experience behavioral abnormalities after levetiracetam administration?

(Question only has to be answered, if you chose **yes** at **question 22**.)

(Please choose only **one** of the following answers.)

- directly after the first levetiracetam intake
- after 1-2 weeks after the first levetiractam intake
- after 3-4 weeks after the first levetiracetam intake
- other time span:

  ____________________________________________________________

1. Did the behavioral abnormalities disappear after discontinuation of levetiracetam?

(Question only has to be answered, if you chose **yes** at **question 22**.)

- Yes
- No
- No discontinuation of levetiracetam
- others:

  ____________________________________________________________

1. Did levetiracetam administration have a positive effect on the behavior of your dog?

- Yes
- No
- If yes,

(Please choose **every** suitable answer.)

- increased activity
- increased energy
- calmer mood
- increased obedience
- other positive effects:

  ________________________________________________

1. Did your dog become seizure-free after levetiracetam administration?

- Yes
- No, but reduced seizure frequency
- No effect
- others:

  ____________________________________________________________

1. When was your dog seizure-free after levetiracetam administration?

(Question has only to be answered, if you chose **yes** at **question 26**.)

(Please choose only **one** of the following answers.)

- directly after the first levetiracetam intake
- after 1-2 weeks after the first levetiractam intake
- after 3-4 weeks after the first levetiracetam intake
- other time span:

  ____________________________________________________________

1. Did your dog ever get a levetiracetam pulse therapy?

(Levetiracetam therapy for minimizing the risk for cluster seizures)

- Yes
- No
- Do not know

1. Did your dog have behavioral abnormalities during levetiracetam pulse therapy?

(Question has only to be answered, if you chose **yes** at **question 28**.)

- Yes
- No
- If yes,

(Please choose **every** suitable answer.)

- - is easily irritable
  - does not like unfamiliar dogs
  - does not like unfamiliar persons
  - learns slowly
  - anxious
  - aggressive
  - is easily distracted
  - hyperactive
  - abnormal behavior as barking without any apparent cause, chasing shadows or staring into space, probably caused by halucinations
  - shows attention seeking behavior towards its owner or other persons
  - decreased interest in activities
  - depressive
  - is agitated if disturbed from sleep
  - None of these, but:

________________________________________________

**Evaluation of behavior factors BEFORE levetiracetam administration**

Answer this section regarding your dogs` behavior **BEFORE** levetiracetam administration.

1. Dog acts anxiously or fearful

(Please choose **every** suitable answer.)

- When approached by unfamiliar dogs
- When in new or unfamiliar surroundings
- When an unfamiliar person enters the house
- Toward unfamiliar persons visiting the home
- When there are sudden or unpredicted movements
- In response to sudden or loud noises
- During thunderstorms
- When first exposed to unfamiliar situations
- When approached directly by an unfamiliar persons outside the house
- In response to wind or wind-blown objects
- When left alone in the house or when a particular family member leaves

1. Dog acts aggressively

(Please choose **every** suitable answer.)

- When approached directly by an unfamiliar dog while being walked or exercised on a leash
- Toward unfamiliar dogs visiting the home
- When being handled
- When people or dogs walk past the house
- When approached by people on walk
- When food, bones, or toys are taken away
- When being corrected or punished
- When approached while eating
- When a familiar person enters the house
- When a stranger enters the house

1. Does your dog ever bite or tried to bite

(Please choose **every** suitable answer.)

- other dogs
- unfamiliar people

1. Dog is seen

(Please choose **every** suitable answer.)

- Barking without any apparent cause
- Chasing light spots or shadows
- Aimlessly pacing or wandering
- Staring into space

1. Dog

(Please choose **every** suitable answer.)

- Is agitated if disturbed from sleep
- Shows reduced interest in activities
- Seems to be less joyful

1. Dog

(Please choose **every** suitable answer.)

- Displays a strong attachment for a particular member of the household
- Tends to follow a member of the household from room to room around the house
- Tends to sit close or in direct contact to a member of the household
- Tends to nudge, nuzzle or paw a member of the household for attention
- Becomes agitated when a member of the household shows affection to a different person or animal

1. Dog

(Please choose **every** suitable answer.)

- Returns immediately when called while off leash
- Obeys a sit command immediately
- Obeys a stay command immediately
- Will fetch or attempt to fetch sticks, balls, and other objects
- Seems to attend to or listen closely to everything the owner says or does
- Is slow to respond to correction or punishment
- Is slow to learn new tricks or tasks

1. Dog

(Please choose **every** suitable answer.)

- Is easily distracted by interesting smells
- Is easily distracted by interesting noises
- Is easily distracted by interesting sights

1. Dog overreacts or is excitable

(Please choose **every** suitable answer.)

- When a member of the household returns after a brief absence
- When playing with a member of the household
- When the doorbell rings
- Just before being taken for a walk
- Just before being taken on a car trip
- When visitors arrive at its home

**Evaluation of behavior factors AFTER levetiracetam administration**

Answer this section regarding your dogs` behavior **AFTER** levetiracetam administration.

1. Dog acts anxiously or fearful

(Please choose **every** suitable answer.)

- When approached by unfamiliar dogs
- When in new or unfamiliar surroundings
- When an unfamiliar person enters the house
- Toward unfamiliar persons visiting the home
- When there are sudden or unpredicted movements
- In response to sudden or loud noises
- During thunderstorms
- When first exposed to unfamiliar situations
- When approached directly by an unfamiliar persons outside the house
- In response to wind or wind-blown objects
- When left alone in the house or when a particular family member leaves

1. Dog acts aggressively

(Please choose **every** suitable answer.)

- When approached directly by an unfamiliar dog while being walked or exercised on a leash
- Toward unfamiliar dogs visiting the home
- When being handled
- When people or dogs walk past the house
- When approached by people on walk
- When food, bones, or toys are taken away
- When being corrected or punished
- When approached while eating
- When a familiar person enters the house
- When a stranger enters the house

1. Does your dog ever bite or tried to bite

(Please choose **every** suitable answer.)

- other dogs
- unfamiliar people

1. Dog is seen

(Please choose **every** suitable answer.)

- Barking without any apparent cause
- Chasing light spots or shadows
- Aimlessly pacing or wandering
- Staring into space

1. Dog

(Please choose **every** suitable answer.)

- Is agitated if disturbed from sleep
- Shows reduced interest in activities
- Seems to be less joyful

1. Dog

(Please choose **every** suitable answer.)

- Displays a strong attachment for a particular member of the household
- Tends to follow a member of the household from room to room around the house
- Tends to sit close or in direct contact to a member of the household
- Tends to nudge, nuzzle or paw a member of the household for attention
- Becomes agitated when a member of the household shows affection to a different person or animal

1. Dog

(Please choose **every** suitable answer.)

- Returns immediately when called while off leash
- Obeys a sit command immediately
- Obeys a stay command immediately
- Will fetch or attempt to fetch sticks, balls, and other objects
- Seems to attend to or listen closely to everything the owner says or does
- Is slow to respond to correction or punishment
- Is slow to learn new tricks or tasks

1. Dog

(Please choose **every** suitable answer.)

- Is easily distracted by interesting smells
- Is easily distracted by interesting noises
- Is easily distracted by interesting sights

1. Dog overreacts or is excitable

(Please choose **every** suitable answer.)

- When a member of the household returns after a brief absence
- When playing with a member of the household
- When the doorbell rings
- Just before being taken for a walk
- Just before being taken on a car trip
- When visitors arrive at its home

**Supplementary Figure 2:** Questionnaire of the study
